# Batch Processing through Particle Swarm Optimization for Target Motion Analysis with Bottom Bounce Underwater Acoustic Signals †

**DOI:** 10.3390/s20041234

**Published:** 2020-02-24

**Authors:** Raegeun Oh, Taek Lyul Song, Jee Woong Choi

**Affiliations:** 1 Department of Marine Science & Convergence Engineering, Hanyang University ERICA, Ansan 15588, Korea; rgoh@hanyang.ac.kr; 2 Department of Electronic Systems Engineering, Hanyang University ERICA, Ansan 15588, Korea

**Keywords:** target motion analysis, bottom bounce path, ray tracing, particle swarm optimization

## Abstract

A target angular information in 3-dimensional space consists of an elevation angle and azimuth angle. Acoustic signals propagating along multiple paths in underwater environments usually have different elevation angles. Target motion analysis (TMA) uses the underwater acoustic signals received by a passive horizontal line array to track an underwater target. The target angle measured by the horizontal line array is, in fact, a conical angle that indicates the direction of the signal arriving at the line array sonar system. Accordingly, bottom bounce paths produce inaccurate target locations if they are interpreted as azimuth angles in the horizontal plane, as is commonly assumed in existing TMA technologies. Therefore, it is necessary to consider the effect of the conical angle on bearings-only TMA (BO-TMA). In this paper, a target conical angle causing angular ambiguity will be simulated using a ray tracing method in an underwater environment. A BO-TMA method using particle swarm optimization (PSO) is proposed for batch processing to solve the angular ambiguity problem.

## 1. Introduction

Acoustic signals are used to indirectly obtain information about objects located underwater. Most passive sonar systems use multiple hydrophones in an array for enhanced performance. A horizontal line array (HLA), used for detecting the azimuth angle of an underwater target, receives acoustic signals with a high signal to noise ratio from designated directions using a beamforming technique. If the target signal intensity is high enough in a designated direction, the target direction is detected. The estimated target direction is represented as a conical angle that indicates the direction of the incoming signal measured by the HLA. Unfortunately, it is impossible to distinguish between up and down or right and left from the conical angle. This is called the cone of ambiguity [1]. 

Sequential processing and batch processing algorithms are used to estimate the target’s state, including position and velocity, through bearings-only target motion analysis (BO-TMA). There exist several conventional sequential processing algorithms, including the extended Kalman filter [2], the pseudo-measurement filter [3], and the modified gain extended Kalman filter [4]. In addition to these filters, particle filter approaches [5] and random finite set approaches [6,7,8] have been recently introduced. If sufficient computational performance is achieved, sequential processing is suitable for implementation in real time systems. However, good sequential estimation results require small errors in the initial state estimates, and a batch processing algorithm is used for this purpose. Batch processing delivers stable initial values, even though it is not designed to operate in real time because it requires a batch of stored measurements. Robust target localization performance is expected if both types of algorithms are employed properly [9]. 

In most of the previous studies on sonar systems [2,3,6,7,8,9], it is assumed that the received signal arrives at the HLA through the horizontal plane when the distance between the observer and target is large, or when the observer and the target are located at equal depths. Therefore, the cone of ambiguity of the HLA is simplified to left/right ambiguity, which can be easily addressed through a ship maneuver. However, eigenray tracing results show that the received signal can arrive at the HLA with a high elevation angle, especially along a bottom bounce path [10]. The studies in [11,12] consider the elevation angles in BO-TMA for different sensor depths between the observer and the target. They treat only direct paths without considering the reflection of the ray from the waveguide boundaries (i.e., sea surface and bottom) or the refraction of the ray from the vertical sound speed profile. However, bottom bounce paths, which are generated from the reflection of acoustic waves at the ocean bottom, can produce inaccurate target bearings [13] that affect BO-TMA results.

The ray tracing method [14] is used to calculate the elevation angle due to the refraction and reflection of sound waves in underwater waveguides. This method describes the path of each ray as sound waves propagating through the underwater waveguide. In particular, it is possible to calculate the eigenray [15], which represents the path of a ray that propagates from the source to the receiver. The elevation angle of the target signal can be simulated through eigenray tracing, and the conical angle can be calculated using the azimuth angle and the elevation angle.

In this paper, a study is based on the published conference paper [16] and it is conducted to confirm the observability of TMA using the conical angle including the elevation angle of the path reflected from the bottom interface for a given scenario. A discrete target dynamic equation is established with the target state vector, and the conical angle measurement is obtained from the relative geometry of the observer and the target using the ray tracing method in Section 2. Section 3 presents a method of converting the conical angle into a bearing line in Cartesian coordinates using knowledge of the ocean environment (i.e., bottom bathymetry and a sound speed profile). Additionally, a BO-TMA using the particle swarm optimization (PSO) algorithm is proposed. In Section 4, simulation results for the BO-TMA are analyzed using ray tracing. Finally, a summary and conclusion are given in Section 5.

## 2. Problem Formulation

### 2.1. Dynamic Model

The target state vector at the discrete time instance k, 1≤k≤K, is defined as:

(1) $$X_{s}\left(k\right)=\left[p_{xs}\left(k\right),\;p_{ys}\left(k\right),\;v_{xs}\left(k\right),\;v_{ys}\left(k\right)\right],$$

(2) $$U_{s}\left(k\right)=\left[u_{xs}\left(k\right),\;u_{ys}\left(k\right)\right],$$

where $p_{xs}\left(k\right)$ and $p_{ys}\left(k\right)$ are the target locations in Cartesian coordinates. Here, the x-axis indicates East and the y-axis indicates North. Additionally, $v_{xs}\left(k\right)$ and $v_{ys}\left(k\right)$ are the target velocities for each direction, and $u_{xs}\left(k\right)$ and $u_{ys}\left(k\right)$ are the target accelerations. The observer state vector is similarly defined as:

(3) $$X_{o}\left(k\right)=\left[p_{xo}\left(k\right),\;p_{yo}\left(k\right),\;v_{xo}\left(k\right),\;v_{yo}\left(k\right)\right],$$

(4) $$U_{o}\left(k\right)=\left[u_{xo}\left(k\right),\;u_{yo}\left(k\right)\right],$$

where the subscript o indicates the observer. Then, the discrete-time system state equation can be described by:

(5) $$X_{i}\left(k+1\right)=FX_{i}^{T}\left(k\right)+GU_{i}^{T}\left(k\right),$$

where $X_{i}$ and $U_{i}$ are the state vectors of the target (when i=s) and the observer (when i=o), and control input, respectively. The superscript T denotes a transpose. The state transition matrix F and input coefficient matrix G are defined, respectively, as:

(6) F=[I2ΔtI202I2],G=[Δt22I2ΔtI2],

where $I_{2}$ is the 2-dimensional identity matrix, $0_{2}$ is the 2×2 zero matrix, and Δt is the time interval. For system observability, we assume that the sensor outmaneuvers the target while the target is moving with a constant velocity [17]. 

**Figure 1:**
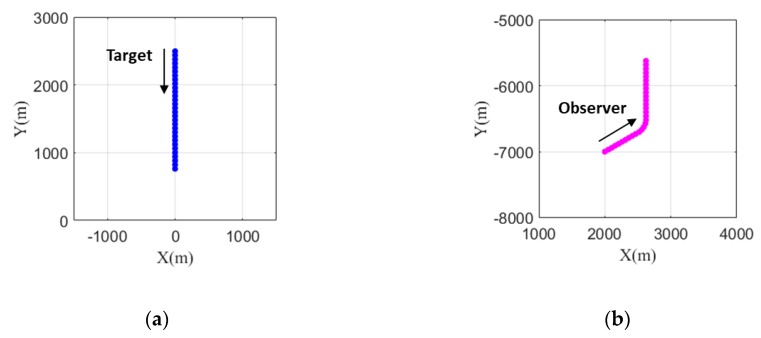
Trajectories of (**a**) the target and (**b**) the observer in the horizontal plane.

The horizontal plane trajectories of the target and the observer located at equal depths of 200 m are shown in Figure 1. The total simulation time is 580 s with a sampling period of 20 s so that the total number of scans is 30. The initial state vector of the target, $X_{s}\left(1\right),$ is [0 m, 2500 m, 0 m/s, −3 m/s] with zero acceleration over the simulation time. The initial state vector of the observer, $X_{o}\left(1\right),$ is [2000 m, −7000 m, 2.6 m/s, 1.5 m/s]. To ensure system observability, the course of the observer is changed once from 60 to 0° via lateral acceleration starting at 200 s. The bearing change rate is 0.6° per second. The distance between the observer and the target is decreased from a maximum distance of 9.7 km to a minimum distance of 6.9 km. 

### 2.2. Measurement Model

Conventional TMA assumes that the target information obtained from passive line array sonar consists of only the azimuth angle in the horizontal plane, neglecting bottom bounce signals to avoid conical angle ambiguity. In this case, the azimuth angle measured from the north axis, $\varphi_{n}\left(k\right)$, is expressed as:

(7) $$\varphi_{n}\left(k\right)=atan2\left(p_{xs}\left(k\right)-p_{xo}\left(k\right),\;\;p_{ys}\left(k\right)-p_{yo}\left(k\right)\right),$$

where atan2(x,  y) denotes a four-quadrant arctangent function that describes the angle between the position of the target and the north axis (positive y-axis). The azimuth angle from the north axis, $\varphi_{n}\left(k\right),$ is converted to the azimuth angle from the direction of the HLA, $\varphi_{l}\left(k\right)$, by subtracting the heading angle of the HLA, $c_{o}\left(k\right)$, at each scan time k:

(8) $$\varphi_{l}\left(k\right)=\varphi_{n}\left(k\right)-c_{o}\left(k\right).$$

**Figure 2:**
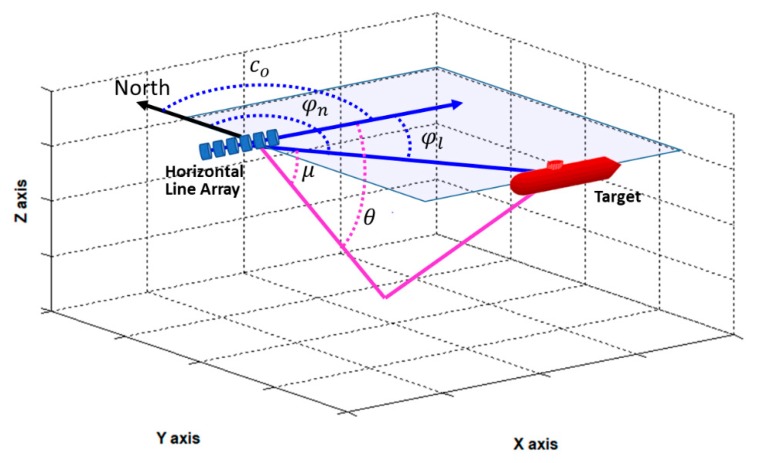
Geometry between observer and target. $\varphi_{l}$ and $\varphi_{n}$ are the azimuth angles from due north and the direction of the HLA, respectively. $c_{o}$, θ, and μ are heading angle of the HLA, conical angle, and elevation angle in the vertical plane, respectively.

In this paper, BO-TMA along with a ray tracing method is used to achieve accurate estimation of target localization in environments with conical angle ambiguity. The conical angle, θ(k), is expressed as:

(9) $$\theta \left(k\right)=cos^{-1}\left(cos\left(\varphi_{l}\left(k\right)\right)\;\times cos\left(\mu \left(k\right)\right)\right)+v\left(k\right),$$

where μ(k) is the elevation angle in the vertical plane, and v(k) is the measurement noise modeled as zero mean Gaussian noise with standard deviation $\sigma_{m}$. The sign of θ(k) is unknown from Equation (9), and the conical angle indicates the magnitude of the angle measured from the heading direction of the line array. Thus, the inability to know the exact direction of the arriving signal is known as left/right ambiguity. Various angles used in this paper are shown in Figure 2. $\varphi_{l}$ and $\varphi_{n}$ are the azimuth angles from due north and the direction of the HLA, respectively. $c_{o}$, θ, and μ are heading angle of the HLA, conical angle, and elevation angle in the vertical plane, respectively.

A ray tracing method is used to estimate the elevation angle of the target signal in Equation (9). In the ocean, propagation paths of acoustic rays are strongly affected by sound speed profile and bottom bathymetry. These environmental data can be obtained through measurements, from a database, or from an ocean prediction model. In this study, a scenario is constructed that assumes a simple environment. The bathymetry is assumed to be flat with a depth of 2000 m. The sound speed profile C(z) in water is assumed to follow Munk’s sound speed profile and is given by [18]:

(10) $$C\left(z\right)=C_{0}\left[1.0+\epsilon \left\{e^{-\eta }-\left(1-\eta \right)\right\}\right],$$

where z is depth, and $C_{0}$ is a reference sound speed equal to 1500 m/s as the sound speed at the depth of channel axis $z_{C}$ ($z_{C}=400\;m$), $\eta =2\left(z-z_{C}\right)/z_{C}$ is a dimensionless depth relative to the channel axis, and the perturbation coefficient ϵ is equal to $7.4\times 10^{-3}$. 

**Figure 3:**
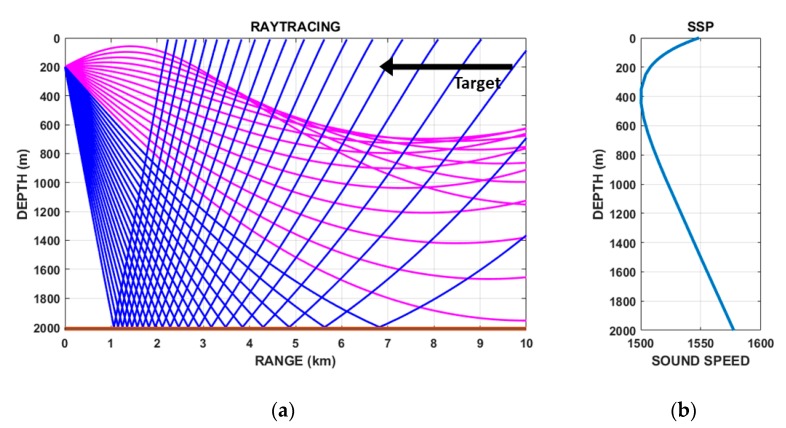
(**a**) Ray paths predicted by ray tracing method based on (**b**) Munk’s sound speed profile. Direct and bottom bounce paths are plotted with magenta and blue lines, respectively.

The ray paths predicted by the ray tracing method using Munk’s sound speed profile are shown in Figure 3. Although ray tracing was conducted based on observer position, the ray tracing results obtained for opposite directions are the same due to the reciprocity of ray diagrams [14]. In addition, the ray tracing results for all azimuth angles are the same because it is assumed that acoustical ocean parameters are independent of azimuth angle. It is shown in this scenario that only bottom reflected paths exist between the target and the observer, and a direct path from the target does not exist. The elevation angle of the bottom bounce path was calculated by ray tracing to be between 23 and 29° at a target distance of 9.7―6.9 km. 

**Figure 4:**
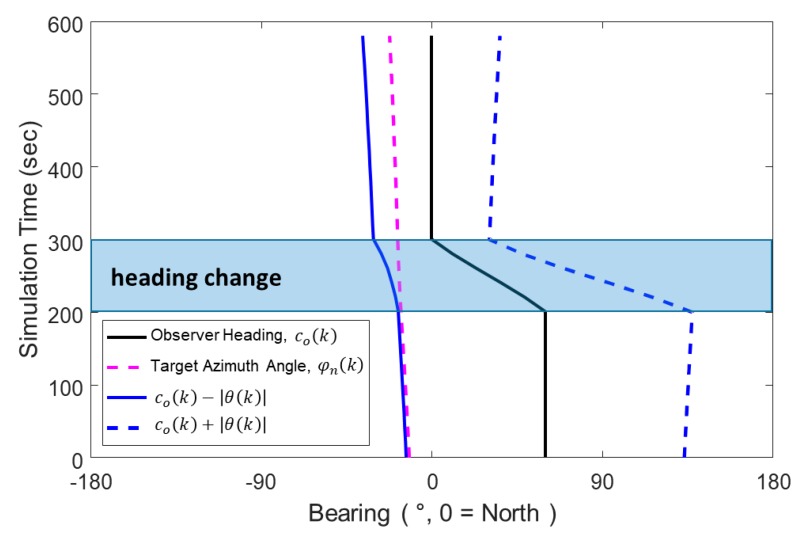
Bearing-time records (BTRs) of the scenario.

Figure 4 shows the simulation results of the bearing measurements from 30 scans over same time period, which is known as BTR (Bearing-Time Record). The red dashed line that represents the azimuth angle from the north axis $\varphi_{n}\left(k\right)$ was plotted as additional information for assessing the bearing error compared to the conical angle of the bottom bounce path. The bearing error is defined as the difference between $\varphi_{l}\left(k\right)$ and +θ(k) or its mirror angle −θ(k) due to conical angle ambiguity. Figure 4 contains the time histories of $c_{o}\left(k\right)$ (the observer heading angle), $\varphi_{n}\left(k\right)$ (the true target azimuth angle), $c_{o}\left(k\right)+\left|\theta \left(k\right)\right|$, and $c_{o}\left(k\right)-\left|\theta \left(k\right)\right|$ (two possible bearing angles for TMA that stem from the bottom bounce path). The right/left ambiguity in the horizontal plane is shown in Figure 4 and can be resolved by comparing the histories of $c_{o}\left(k\right)+\left|\theta \left(k\right)\right|$ and $c_{o}\left(k\right)-\left|\theta \left(k\right)\right|$. The history of $c_{o}\left(k\right)-\left|\theta \left(k\right)\right|$ has smaller variations than that of $c_{o}\left(k\right)+\left|\theta \left(k\right)\right|$. Note that these two angle histories correspond to the history of the true azimuth angle $\varphi_{n}\left(k\right)$. The history of $\varphi_{n}\left(k\right)$ in Figure 4 shows small variations for the entire period that includes the times before and after the observer maneuver, which implies that $c_{o}\left(k\right)-\left|\theta \left(k\right)\right|$ rather than $c_{o}\left(k\right)+\left|\theta \left(k\right)\right|$ should be applied as the bearing history for this scenario in TMA. From the selection process, the correct sign of θ(k) in Equation (9) for this scenario is negative. However, even after choosing the bearing history with the correct sign of θ(k), $c_{o}\left(k\right)-\left|\theta \left(k\right)\right|$ still contains bearing error when compared to the true azimuth angle history $\varphi_{n}\left(k\right)$. Figure 4 shows that this error is ~1° before the observer maneuver and ~13° after the maneuver. This discrepancy is due to μ(k), the elevation angle of the bottom bounce path. Conventional TMA methods for target localization cannot avoid localization errors resulting from bearing errors. Therefore, a new TMA method that accounts for the bottom bounce path is needed.

## 3. Target Motion Analysis with Bottom Bounce Path

### 3.1. Bearing Lines of Bottom Bounce Path

Bearing error is due to the elevation angle μ(k) of the bottom bounce path, which is unknown even after selection of the correct sign of θ(k). In this study, the bearing line in Cartesian coordinates is introduced. Define the i-th expected azimuth angle ${\hat{\varphi }}_{l}\left(k,\;i\right)$ for 1≤i≤I, which represents a possible target azimuth angle relative to the heading direction of the HLA. According to Equation (9), ${\hat{\varphi }}_{l}\left(k,\;i\right)$ must lie within the range between zero and the conical angle θ(k), and then the elevation angle $\hat{\mu }\left(k,\;i\right)$ can be estimated as:

(11) $$\hat{\mu }\left(k,\;i\right)=cos^{-1}\left(\frac{cos\left(\theta \left(k\right)\right)}{cos\left({\hat{\varphi }}_{l}\left(k,\;i\right)\right)}\right).$$

**Figure 5:**
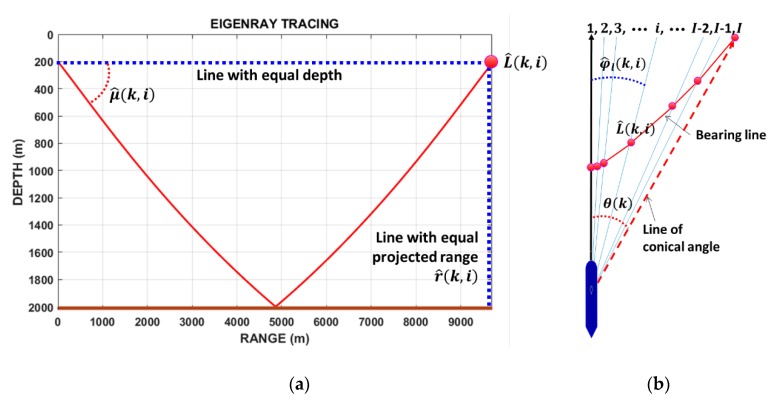
(**a**) Eigenray tracing result conducted to determine the expected target range. The distance at which the ray arrives at an expected target depth after bottom reflection becomes the estimated target range in ${\hat{\varphi }}_{l}\left(k,\;i\right)$ direction. (**b**) Top-view illustration showing the line of conical angle and bearing line. For *k*-th scan, the line connecting *I* possible target positions estimated using the eigenray tracing is a bearing line (red line in figure).

The sign of ${\hat{\varphi }}_{l}\left(k,\;i\right)$ is equal to the sign of θ(k). For each ${\hat{\varphi }}_{l}\left(k,\;i\right)$, ray tracing for the ray launched at an angle of $\hat{\mu }\left(k,\;i\right)$ from the observer position is conducted to find the range $\hat{r}\left(k,\;i\right)$ of target location if it exists in the direction of ${\hat{\varphi }}_{l}\left(k,\;i\right)$ (Figure 5a). Since the target depth was assumed to be 200 m, the distance at which the ray arrives at a water depth of 200 m after bottom reflection becomes the target range in the ${\hat{\varphi }}_{l}\left(k,\;i\right)$ direction. This process is repeated i=I times (Figure 5b). In this study, the expected azimuth angle was varied every 0.5°. Accordingly, |θ(k)| divided by 0.5° was used to determine the value of I for each scan k. 

For the k-th scan, I possible target positions in the horizontal plane corresponding to every ${\hat{\varphi }}_{l}\left(k,\;i\right)$ are connected in a line, which is defined as a *bearing line* in this paper. The possible target position vector in Cartesian coordinates with ${\hat{\varphi }}_{l}\left(k,\;i\right)$ and $\hat{r}\left(k,\;i\right)$ is denoted as:

(12) $$\hat{L}\left(k,\;i\right)=\left[{\hat{p}}_{xl}\left(k,\;i\right),\;{\hat{p}}_{yl}\left(k,\;i\right)\right].$$

**Figure 6:**
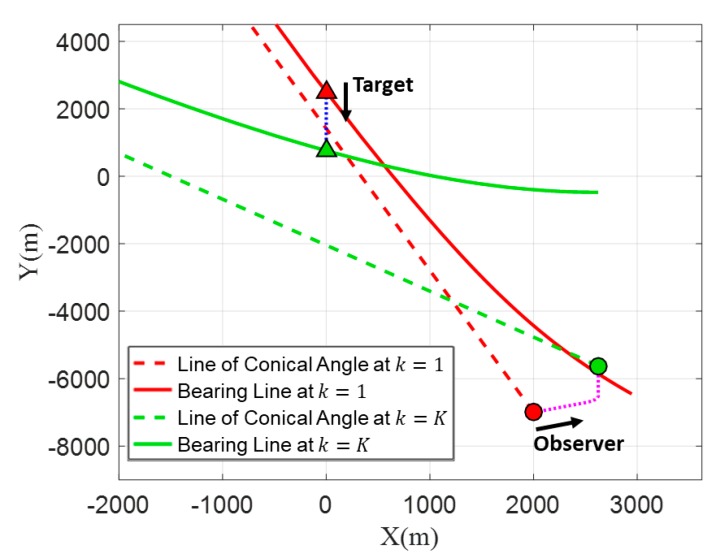
Bearing lines (solid lines) and lines of conical angles (dashed lines) at k=1 and k=K.

Figure 6 is drawn in the horizontal plane and it shows the bearing lines corresponding to k=1 and k=K. The lines (denoted by line of conical angle) indicating the measured conical angle θ(k) in the horizontal plane for k=1 and k=K. If the elevation angle is not considered, as in previous studies, the bearing line is displayed as a straight line. However, the bearing line $\hat{L}\left(k,\;i\right)$ is displayed as a curved line when the elevation angle is considered. Conventional batch estimation methods for TMA utilize the conical angles to determine the initial target states, while the proposed TMA method utilizes the bearing lines. The objective of the proposed TMA method is to find the optimal initial position and velocity of the target based on the bearing lines in Cartesian coordinates using the PSO algorithm to minimize the objective function.

### 3.2. Particle Swarm Optimization

The PSO algorithm is a stochastic optimization algorithm used to find the optimal positions of particles and is based on the social behavior of animals moving in flocks [19,20]. In BO-TMA studies, each particle representing an estimated initial target state vector consists of four elements: the positions and velocities in the x and y directions. First, at k=1, the particles are uniform, randomly spread along the bearing line within the target-observer distance from 1 to 30 km. A specific velocity vector, which is randomly selected in the range of $0\left|\hat{v}\right|\;\;10$ m/s, where $\left|\hat{v}\right|=\sqrt{{\left|{\hat{v}}_{x}\right|}^{2}+{\left|{\hat{v}}_{y}\right|}^{2}}$, is assigned to each particle. Then, the position of each particle at the next scan time (k=2) is calculated using the dynamic model shown in Section 2.1 from the position at k=1. In this manner, a total of K positions are determined for each particle, which forms a particle trajectory. After that, the shortest distance between each particle position and the bearing line corresponding to the same scan time number is calculated. This distance is then normalized by the distance between the observer and particle position at each scan time to avoid excessive convergence to local optima, which happens because distance error increases as the distance between the observer and the particle increases. Finally, the normalized distance errors for all K particle positions are summed to obtain an objective function $J^{m}$ for the m-th particle, which is expressed as:

(13) $$J^{m}=\overset{K}{\underset{k=1}{\sum }}\frac{\begin{array}{c} min\\ i \end{array}\sqrt{{\left({\hat{p}}_{xm}\left(k\right)-{\hat{p}}_{xl}\left(k,\;i\right)\right)}^{2}+{\left({\hat{p}}_{ym}\left(k\right)-{\hat{p}}_{yl}\left(k,\;i\right)\right)}^{2}}}{\sqrt{{\left({\hat{p}}_{xm}\left(k\right)-p_{xo}\left(k\right)\right)}^{2}+{\left({\hat{p}}_{ym}\left(k\right)-p_{yo}\left(k\right)\right)}^{2}}},$$

where ${\hat{p}}_{xm}\left(k\right)$ and ${\hat{p}}_{ym}\left(k\right)$, respectively, are the positions in the x and y directions of the m-th particle at scan time k; and $p_{xo}\left(k\right)$ and $p_{yo}\left(k\right)$ are the observer positions in the x and y directions at scan time k. The total particle number used here was 200 (Table 1). Since each particle is considered a candidate for the target, the next step is to find the initial state vector of the particle that produces the minimum value of $J^{m}$. In this study, the PSO algorithm was used as an optimization technique to find the optimal target trajectory. In each generation, the best values for the state vectors consisting of the positions and velocities of the particles are evaluated by comparison with state vectors selected during previous generations, and the state change rates of the particles are adjusted based on the experiences of the particles and their companions. The state vectors in the next generation are updated with the sum of the present state vectors and the adjusted state change rates of the particles [20]. The process is expressed as [19]:

(14) $$v_{p}\left(n+1,\;m,\;d\right)=c_{1}v_{p}\left(n,m,d\right)+v_{l}\left(n,m,d\right)+v_{s}\left(n,m,d\right),$$

(15) $$v_{l}\left(n,m,d\right)=c_{2}r_{1}\left\{x_{pl}\left(m,d\right)-x_{p}\left(n,m,d\right)\right\},$$

(16) $$v_{s}\left(n,m,d\right)=c_{3}r_{2}\left\{x_{ps}\left(d\right)-x_{p}\left(n,m,d\right)\right\},$$

(17) $$\mathrm{and}\;x_{p}\left(n+1,m,d\right)=x_{p}\left(n,m,d\right)+v_{p}\left(n+1,\;m,\;d\right),$$

where $x_{p}\left(n,m,\;d\right)$ represents the state vector of the m-th particle for the n-th generation with dimension d. Dimension d is one of 1, 2, 3, and 4 corresponding to the positions and velocities of the particles at k = 1, that is, ${\hat{p}}_{xm}\left(1\right)$, ${\hat{p}}_{ym}\left(1\right)$, ${\hat{v}}_{xm}\left(1\right)$, and ${\hat{v}}_{ym}\left(1\right)$, respectively. In addition, $v_{p}\left(n,m,\;d\right)$ represents the state change rates of the particles for $x_{p}\left(n,m,\;d\right)$. Finally, $v_{l}\left(n,m,\;d\right)$ and $v_{s}\left(n,m,\;d\right)$ are the local state change rate and the social state change rate for the m-th particle, respectively. The local state vector $x_{pl}\left(m,\;d\right)$ is the best state vector of the m-th particle obtained from the first generation to the n-th generation, and the social state vector $x_{ps}\left(d\right)$ is the best state vector of the particle with the smallest $J^{m}$ of all particles up to the n-th generation. In the above equations, $c_{1}$, $c_{2}$, and $c_{3}$ are acceleration weight constants determined empirically through many trial runs to be 0.73, 0.1, and 0.2, respectively. Random numbers $r_{1}$ and $r_{2}$ are selected in the range between 0 and 1. The process is iterated until the state vector of each particle converges to the best state vector that satisfies the minimum position errors. In our case, the generation is terminated when the standard deviations of the positions, $\sigma_{p}$, and velocities, $\sigma_{v}$, of 200 particles converge to values less than 100 m and 0.2 m/s, respectively. Finally, the trajectory of the particle with the best state vector is selected as the target trajectory.

## 4. Simulation Result

**Figure 7:**
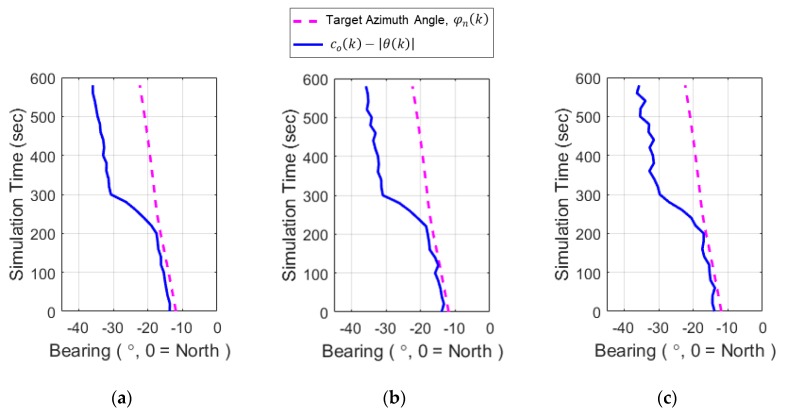
The BTRs for conical angle measurements including Gaussian measurement error with zero mean and standard deviation of (**a**) 0.2, (**b**) 0.4, and (**c**) 0.6°.

For the observability test, it was assumed that the water depth was 2000 m and the bottom topography was flat. The conical angle was calculated using the azimuth and elevation angle of the acoustic ray path between the target and the observer. Munk’s sound speed profile was used for ray tracing to calculate the elevation angle. To test the applicability of batch processing using the PSO algorithm proposed in this paper, it was assumed that Gaussian noise with zero mean and standard deviation $\sigma_{m}$ was included in the conical angle measurements. Three values of $\sigma_{m}$ (0.2, 0.4, and 0.6°) were considered for comparison purposes. For this scenario, the conical angle was estimated to change at a rate of approximately 0.5°/scan except during the period in which the observer heading changed. Figure 7 shows the histories of conical angles with measurement errors corresponding to three different standard deviations.

**Figure 8:**
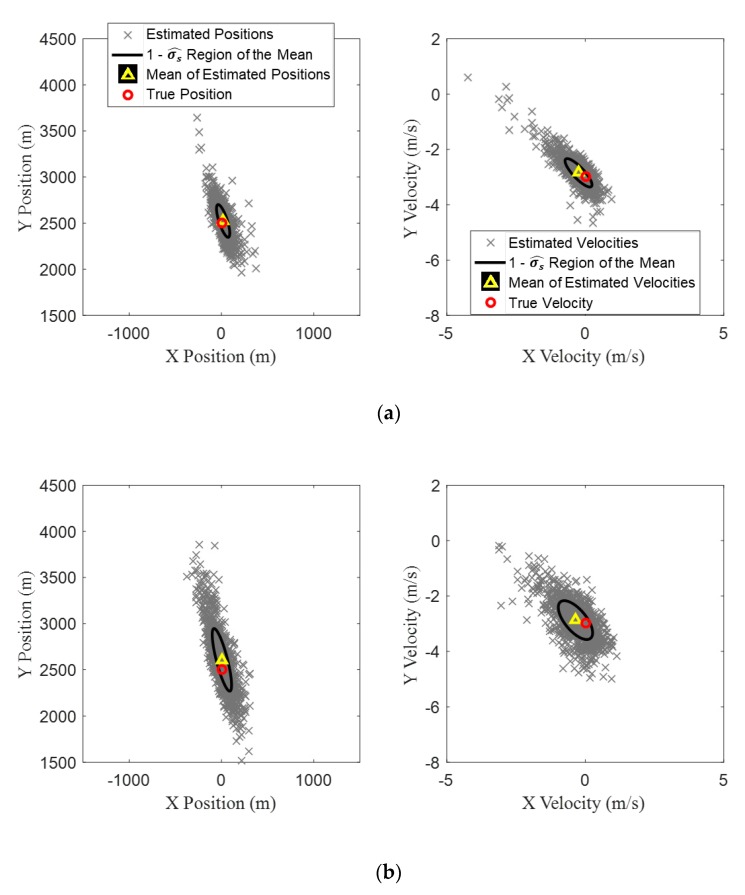

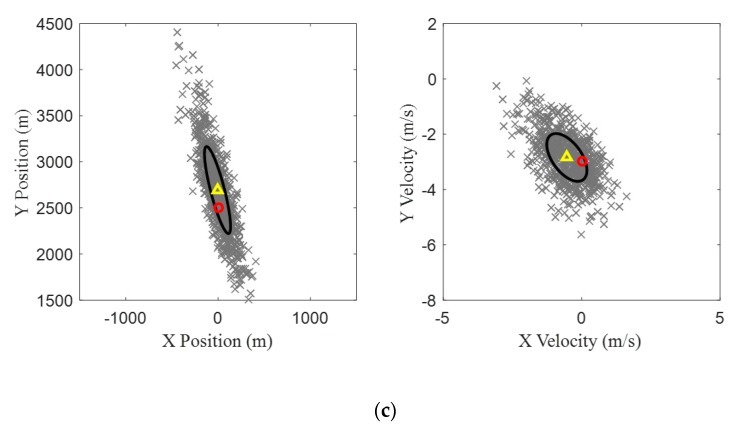
The distribution of initial states estimated using TMA for 1000 random runs for standard deviations of zero mean Gaussian measurement errors of (**a**) 0.2, (**b**) 0.4, and (**c**) 0.6°. The true initial state vector of the target is [0 m, 2500 m, 0 m/s, −3 m/s]. The left column shows the initial target position estimates, and the right shows target velocity estimates. The true initial state vector of the target and the mean of estimated state vectors are indicated by red circles and yellow triangles, respectively. The regions within one standard deviation of the mean are indicated by black ellipses.

One thousand random runs were generated for each of the three standard deviations of the conical angle measurement errors, and TMA was carried out for each run. The results are shown in Figure 8, in which the left column shows the scatter plots for the estimates of initial target position for the 1000 runs, and the right column shows the scatter plots of target velocity. For the different standard deviations, the mean values of the estimated initial state vectors and their variances are listed in Table 2. The results show that, as the standard deviation of the measurement error increases, the distribution of the initial state vector obtained from the proposed BO-TMA becomes wider. In particular, as the measurement error increases, the estimated positions of the target tend to spread wider along the bearing line at k=1, which is reasonable because the particles were spread along the bearing line at k=1. The mean value of the initial state vector estimated for the standard deviation of 0.2° (marked by a yellow triangle in the figure) has the best agreement with the true initial target state vector (marked by red circle), and as the standard deviation increases, the difference increases slightly. However, the mean values for the three cases are still in good agreement with the true values.

**Figure 9:**
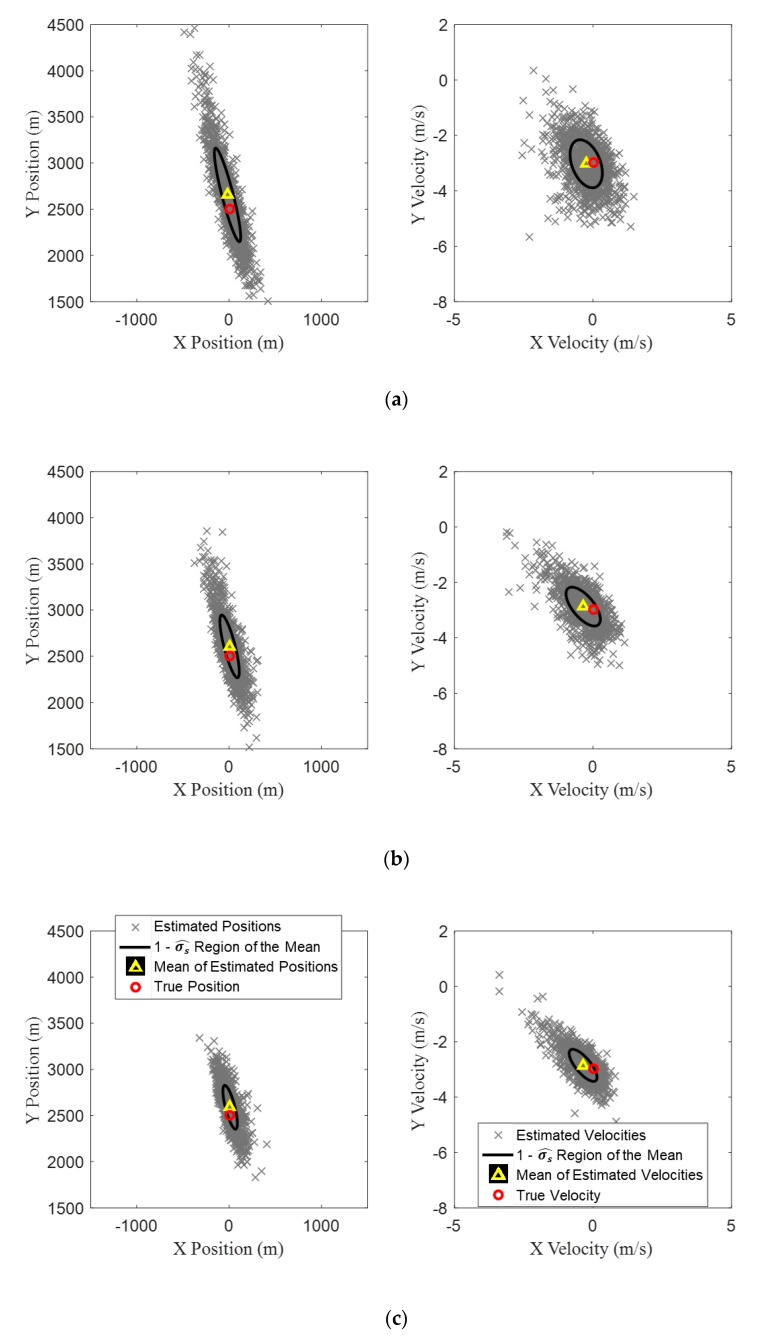
The distribution of initial states estimated using TMA for 1000 random runs with standard deviations of zero mean Gaussian measurement error of 0.4° with the measurement numbers of (**a**) 15, (**b**) 30, and (**c**) 60.

To investigate the accuracy of the TMA results with increasing the number of scans k, the processes were repeated with the scan numbers of 15, 30, and 60 which correspond to the sampling periods of 40, 20, and 10 s, respectively. The standard deviation of the conical angle measurements were assumed to be 0.4°. The estimation results of the initial target state vector with the three scan numbers are shown in Figure 9, and the resulting mean values and variances are listed in Table 3. Figure 9 and Table 3 indicate that more frequent collection of conical angle measurements achieves more accurate TMA results with increased expense of computational resources.

As shown in Figure 7, the bearing errors due to elevation angle after the observer maneuver are larger than 10°. Conventional TMA methods produce large localization errors in environments dominated by acoustic rays being strongly reflected or refracted up and down. However, the proposed BO-TMA method using ray tracing shows good localization performance in such environments, which implies that the proposed TMA method is a more effective tool for increasing solution accuracy in real underwater applications, especially in waveguide environments where bottom bounce paths are dominant.

## 5. Summary and Conclusion

In this paper, a BO-TMA algorithm using a ray tracing method is proposed to accurately consider the conical angles generated by bottom bounce paths. The 3-dimensional conical angle information was converted to bearing lines in a 2-dimensional plane using a ray tracing method. Then, the PSO algorithm was carried out based on the constructed bearing lines to find optimal target state vectors.

The BO-TMA method using ray tracing and the PSO algorithm proposed in this paper is summarized below.

(1) Convert the conical angles of the bottom bounce path to a bearing line using the ray tracing technique. Set the generation number n=1.

(2) Initialize particles with the bearing line at k=1. Uniform, randomly spread particles on the bearing line and assign velocities randomly selected in the range $0\leq \left|\hat{v}\right|\leq 10$ m/s.

(3) For each particle with a four-element state vector, calculate the objective function $J^{m}$ using the particle trajectories and the bearing lines corresponding to k=1,⋯,K. 

(4) Find the particle that produces the minimum value of $J^{m}$.

(5) Generate the next generation particle group by applying the PSO algorithm.

(6) Go to Step (3), and then iterate the process.

(7) Terminate the iteration when the state vectors of the particles reach the termination condition.

In this paper, a ray tracing technique was used to calculate the elevation angle. The conical angle of the target was then calculated based on the estimated elevation angle. Characteristics of the oceanic environment are known, allowing for accurate estimation of elevation angles. However, since the oceanic environment fluctuates temporally and spatially, errors can arise from uncertainty in environmental information. In addition, we assumed that target depth is the same as observer depth. Uncertainty in target depth may also result in target distance errors. Therefore, further research into various target-observer geometries and various ocean environments is required to generalize the results shown in this paper. 

## Figures and Tables

**Table 1:** Particle swarm optimization parameters used to find the optimal initial position and velocity of target.

Parameter	Symbol	Value
Number of particles	m	200
Number of dimensions	d	4
Number of generations	n	$\sigma_{p}<100$ m and $\sigma_{v}<0.2$ m/s
Acceleration weight constants $c_{1}$	$c_{1}$	0.73
Acceleration weight constants $c_{2}$	$c_{2}$	0.1
Acceleration weight constants $c_{3}$	$c_{3}$	0.2
Random number $r_{1}$	$r_{1}$	0―1
Random number $r_{1}$	$r_{2}$	0―1

**Table 2:** The means and variances of the estimated initial state vector [${\hat{p}}_{xm}\left(1\right)$, ${\hat{p}}_{ym}\left(1\right)$, ${\hat{v}}_{xm}\left(1\right)$, ${\hat{v}}_{ym}\left(1\right)$ ] for three values of standard deviation of measurement error.

$\mathrm{Standard}\;\mathrm{Deviation}\;\mathrm{of}\;\mathrm{Measurement}\;\mathrm{Noise},\;\sigma_{m}$	$\mathrm{Mean}\;\mathrm{of}\;\mathrm{Initial}\;\mathrm{Target}\;\mathrm{State}\;\mathrm{Vector},\;\hat{X_{s}},$ [m, m, m/s, m/s]	$\mathrm{Variance}\;\mathrm{of}\;\mathrm{Initial}\;\mathrm{Target}\;\mathrm{State}\;\mathrm{Vector},\;{\hat{\sigma_{s}}}^{2},$ $[m^{2}$ $,\;m^{2}$ $,\;m/s^{2}$ $,\;m/s^{2}]$
0.2°	[18, 2524, −0.2, −2.8]	$\left[71^{2},\;178^{2},\;0.5^{2},\;0.5^{2}\right]$
0.4°	[5, 2607, −0.4, −2.9]	$\left[105^{2},\;340^{2},\;0.6^{2},\;0.7^{2}\right]$
0.6°	[−6, 2693, −0.5, −2.8]	$\left[138^{2},\;473^{2},\;0.7^{2},\;0.9^{2}\right]$

**Table 3:** The means and variances of the estimated initial state vector [${\hat{p}}_{xm}\left(1\right)$, ${\hat{p}}_{ym}\left(1\right)$, ${\hat{v}}_{xm}\left(1\right)$, ${\hat{v}}_{ym}\left(1\right)$] for three different measurement numbers.

Number of Measurements, k	$\mathrm{Mean}\;\mathrm{of}\;\mathrm{Initial}\;\mathrm{Target}\;\mathrm{State}\;\mathrm{Vector},\;\hat{X_{s}},$ [m, m, m/s, m/s]	$\mathrm{Variance}\;\mathrm{of}\;\mathrm{Initial}\;\mathrm{Target}\;\mathrm{State}\;\mathrm{Vector},\;{\hat{\sigma_{s}}}^{2},$ $[m^{2}$ $,\;m^{2}$ $,\;m/s^{2}$ $,\;m/s^{2}]$
15	[−18, 2655, −0.2, −3.0]	$\left[138^{2},\;509^{2},\;0.6^{2},\;0.9^{2}\right]$
30	[5, 2607, −0.4, −2.9]	$\left[105^{2},\;340^{2},\;0.6^{2},\;0.7^{2}\right]$
60	[11, 2590, −0.4, −2.9]	$\left[82^{2},\;236^{2},\;0.5^{2},\;0.6^{2}\right]$
